# Retraction: Resveratrol and Black Tea Polyphenol Combination Synergistically Suppress Mouse Skin Tumors Growth by Inhibition of Activated MAPKs and p53

**DOI:** 10.1371/journal.pone.0215980

**Published:** 2019-04-19

**Authors:** The PLOS ONE Staff

Following the publication of this article [1], the following concerns were raised:

1. Figure 3A panel IV and Figure 3B panel IV appear to contain areas of similarity;
2. Figure 3A panels I, V, and VI appear to contain areas of similarity;
3. Figure 3B, panels I and II contain areas of similarity;
4. Figure 3B, parts of panels V and VI contain areas of similarity;
5. Figure 6 of a previously published article in the *Journal of Food and Chemical Toxicology* [2] by four of the authors and Figure 6 in this article [1] appear to contain similar figures: Figure 6a in [2] appears similar to Figure 6(I) in [1]; Figure 6c in [2] appears similar to Figure 6(III) in [1];
6. Repeated regions of similarity in flow cytometry plots within Fig 6 IV, VI, and II;
7. Figure 2A ERK1/2 (Total) bands in lanes 5 and 6 appear similar when adjusted for brightness/contrast;
8. Figure 2A p38 (Total) panel lanes 2 and 6 appear similar when adjusted for brightness/contrast;
9. Figure 2A ERK1/(P) panel contains vertical discontinuities across all lanes when adjusted for brightness/contrast;
10. Figure 5 p53 contains background irregularities when adjusted for brightness/contrast such that lane 2 appears to have a background different to other lanes.

The authors have been unable to provide any primary data underlying the figures. The authors commented that the regions of similarity in histopathological images arose due to similarity in lesions studied. For the similarities noted in flow cytometry plots, the authors commented that this could have arisen due to similarity in equipment and protocols.

In the absence of the data underlying the figures and in light of the above concerns, the *PLOS ONE* Editors retract the article.

The authors did not comment on the retraction decision.
